# Effects of Cantaloupe (*Cucumis melo*) Melon Seed Flour on Physicochemical Characteristics and Consumer Acceptance of Gluten-Free Cookies

**DOI:** 10.3390/foods14234117

**Published:** 2025-12-01

**Authors:** Sagrario Medina, Roberto Cedillos, Silvia Murillo Miguez, Andrea Muela, Elio Villasmil, Jhunior Marcia, Witoon Prinyawiwatkul

**Affiliations:** 1 Faculty of Technological Sciences, Universidad Nacional de Agricultura, Road to Dulce Nombre 9 de Culmí, Km 215, Barrio El Espino, Catacamas 16201, Olancho, Honduras; sagrariomedina100@gmail.com (S.M.); jmarcia@unag.edu.hn (J.M.); 2 School of Nutrition and Food Sciences, Louisiana State University Agricultural Center, Baton Rouge, LA 70803, USA; rcedil1@lsu.edu (R.C.); smurillomiguez1@lsu.edu (S.M.M.); amuela1@lsu.edu (A.M.); evill15@lsu.edu (E.V.)

**Keywords:** cantaloupe, melon seed flour, upcycling, food waste, sustainability, gluten-free cookies

## Abstract

Cantaloupe melon seeds are a byproduct that can be upcycled for their nutritional value, generating added value, reducing food waste, and supporting food sustainability. This study evaluated the effects of melon seed flour on selected physicochemical and consumer acceptance of gluten-free cookies. Melon seeds were dehydrated at 60 °C for 12 h and ground. Then gluten-free cookies containing varying melon seed flour (20, 40, 60, 80, and 100%) were prepared by mixing the ingredients and baked at 177 °C for 18 min. Color, water activity, proximate composition, and mineral contents of the melon seed flour were measured. Color, water activity, spread factor, and hardness of the five cookie formulations were evaluated. Finally, a randomized block design was used for the consumer test with 90 consumers. Appearance, aroma, flavor, texture, grittiness, and overall liking were evaluated using a 9-point hedonic scale. Also, purchase intent was asked for before and after a sustainability claim. Data were analyzed using an ANOVA and the post hoc Tukey test (*p* < 0.05). The McNemar test was used to test whether there were significant differences in purchase intent before and after a sustainability claim. Melon seed flour had 21.4% protein, 34.93% crude fiber, 3% ash, 4% moisture, and 26.9% fat. Spread factor and a* (color redness) values increased with increasing melon seed flour. On the other hand, the more melon seed flour in cookies, the lower the L* value and water activity. The treatment with 40% melon seed flour had the highest liking score, 6.25. Finally, the sustainability claim significantly increased the positive purchase intent of the cookies. This study demonstrates the potential of cantaloupe melon seed flour as an ingredient in food, such as gluten-free cookies. This practice in the food industry can help increase value and reduce waste in cantaloupe processing.

## 1. Introduction

Food waste is a crucial issue not just for the food industry but also for consumers, given its environmental, social, and economic impacts. “Food waste” is defined as food and the associated inedible parts removed from the human food supply chain [1]. According to UNEP [1], the world wasted an estimated 1.05 billion tons of food in the retail, food service, and household sectors combined in 2022. There are major factors contributing to the food wastes issue; first, the production in the fields due to pests and deficient agricultural practices [2]; second, the efficiency of food processing and packaging stages that impact on yields [1,3]; third, quality standards and overstocking of the retailers and market services [4,5]; finally, consumer behavior including overpurchased amounts of food, improper storage, and lack of meal planning [6].

Cantaloupe melon (*Cucumis melo*) is an important crop and is consumed worldwide for its flavor and health benefits. This fruit is particularly attractive due to its content of sugars, bioactive components, water, vitamin C, and potassium [7]. The global cantaloupe production was approximately 36.5 million metric tons [8]. Depending on the type of melon, the percentage of the seeds is approximately 2% to 15% of the total weight of the melons, indicating a significant byproduct fraction that is often discarded and never reaches a consumer’s plate. Seeds from the juice and fresh-cut produce industries account for the majority of this waste [9,10].

Cookies are a very popular snack that is usually offered as a dessert. They are typically crispy and sweet, made generally with wheat flour, shortening, sugar, and eggs [11]. There are alternative non-wheat cookie options marketed for people who have celiac disease due to their intolerance to gluten proteins. In accordance with Rubio-Tapia et al. [12], by 2010, 1 in every 141 (0.71%) people in the United States had celiac disease. Moreover, the market value of gluten-free products was USD 6.45 billion and is expected to grow at a compound annual growth rate (CAGR) of 9.8% from 2023 to 2030 [13], making it a profitable market to participate in.

Gluten-free products have gained significant popularity in recent years. The use of non-traditional ingredients in gluten-free cookie formulations can provide both unique flavor and acceptable texture qualities that ensure consumer product accessibility for individuals with celiac disease, hence, offering potential marketing advantages [14].

By utilizing the byproducts of the food industry that would otherwise end up in the trash, the industry can generate products with added value while reducing waste sent to the environment [15]. Additionally, leveraging raw materials (e.g., melon seeds) that are not commonly used in the industry may positively impact the company’s profitability [16] and, consequently, its competitiveness in the market.

Even though other studies have tested the inclusion of melon seed flour in cakes or beverages, there have been no studies testing the incorporation of cantaloupe melon seed flour into gluten-free cookies. Our main goal was to maximize the use of cantaloupe melon seed flour as a functional ingredient in gluten-free rice-flour-based cookie formulations. The melon seed flour was the target innovation, not the rice flour; in other words, the control (100% rice flour formulation) was intentionally not used as a gold standard for comparison in our study. Therefore, the aims of this study were to elucidate selected physicochemical characteristics of cantaloupe melon seed flour, to characterize the physicochemical properties of gluten-free cookies containing varying levels of added melon seed flour, and, finally, to evaluate consumer acceptance and purchase intent for these cookies.

## 2. Materials and Methods

### 2.1. Experimental Design

A completely randomized design was implemented for the determination of the physicochemical characteristics of the cookies made with melon seed flour. Five cookie formulations were prepared as described in Table 1. For the consumer acceptance test, a randomized complete block design was used with each panelist being a block.

### 2.2. Cantaloupe Melon Seed Flour Production

The production of cantaloupe melon seed flour was conducted as described by da Cunha et al. [17] with slight modifications. First, the seeds were obtained from a local supermarket; they were waste from the in-store fresh-cut processing. The seeds were washed using tap water to remove excess pulp that remained on the seeds. Afterwards, the seeds were dehydrated in an air-fryer oven (Ninja Foodi Smart XL Pro air Oven, Needham, MA, USA) for 12 h at 60 ± 1 °C. Once dried, seeds were ground (Ninja BN401 Nutri Pro Compact Personal Blender, Needham, MA, USA) for 1 min to obtain a homogeneous flour. Finally, the melon seed flour was stored in glass jars away from sunlight until further analysis.

### 2.3. Physicochemical Characteristics of Cantaloupe Melon Seed Flour

#### 2.3.1. Water Activity

Water activity (a_W_) was determined as described by Carter et al. [18]. The melon seed flour (5 ± 0.1 g) was placed in the Aqualab 4TE (Decagon Devices, Pullman, WA, USA) previously calibrated with a_W_ standards as suggested by the manufacturer at 25 ± 1 °C. The a_W_ was expressed on a scale from 0 to 1.

#### 2.3.2. Color

Color values (L*, a*, and b*) were determined using a colorimeter (Chroma meter CR-410, Konica Minolta, Inc., Osaka, Japan). A 50 g sample of melon seed flour was used for all measurements. The apparatus was previously standardized with a calibration plate as suggested by the manufacturer.

#### 2.3.3. Proximate Composition and Mineral Content

Water content was measured by AOAC 930.15/2005 [19] with slight modifications. A 3-g cantaloupe melon seed flour was placed in an aluminum dish in a preheated convection oven (Model 1370 GM, Sheldon Manufacturing Inc., Cornelius, OR, USA) for 12 h at 105 °C. Afterwards, moisture was calculated based on the water that was removed and expressed in percentage as explained in the following Equation (1):

Water Content (%) = [(Water Extracted/Initial Flour Weight)] × 100(1)

Fat content was determined using the modified method of AOAC 2003.06 [20]. A 10-g melon seed flour was completely dehydrated using the method previously described above. The dried sample was placed in a 250-mL Soxhlet extraction apparatus (Wheaton, Millville, NJ, USA) submerged in 200 mL of hexane for 2 h. Afterwards, the mixture of solvent and fat was processed in the rotary evaporator (Rotary Evaporator RE500, Yamato Scientific America, Inc., Santa Clara, CA, USA) at 30 ± 1 °C under vacuum conditions to separate the hexane from the fat phase. Subsequently, it was placed in a preheated convection oven (Model 1370 GM, Sheldon Manufacturing Inc., Cornelius, OR, USA) for 24 h at 60 °C. Finally, the fat residue was weighed, and the fat percentage was determined following Equation (2) below.

Fat (%) = [(Fat Extracted/Initial flour sample)] × 100(2)

The AOAC 990.03 [21] method was used to determine protein content by combustion analysis using a protein/nitrogen analyzer (Flash 2000, Thermo Fisher Scientific, Waltham, MA, USA). A 30-mg sample was subject to the following conditions: oxygen 40 PSI, helium 40 PSI, the lift furnace heat up to 950 °C, the right furnace at 840 °C, oven heat at 50 °C, timing of the carrier 140 mL/min, oxygen at 300 mL/min, and the reference at 10 mL/min, running time of 340 s, sampling delay of 10 s, and oxygen injection end of 15 s. The instrument was previously calibrated according to the manufacturer’s suggestion, and the protein content was expressed as a percentage. The conversion factor of 6.25 was used to transform the mass of nitrogen into the mass of protein.

Ash content was determined according to Marshall [22] with slight modifications. A 3 ± 0.1 g sample was placed in a crucible and placed inside a muffle furnace. The samples were left to burn at 550 °C for 24 h. Afterwards, the muffle was turned off to let it cool down. Finally, after 24 h, the ash was weighed. Ash percentage was determined following Equation (3).

Ash (%) = [(Ashes residue weight/Initial sample weight)] × 100(3)

For mineral analysis, the AOAC 985.01 method was followed [23]. First, 0.50 ± 0.01 g of sample was placed in a 100-mL volumetric flask (previously rinsed with a 50:50 *v*/*v* mixture of nitric acid and 6 N hydrochloric acid, then washed with deionized water). Then, 20 mL of a 7:1 *v*/*v* mixture of nitric acid and perchloric acid was added, and the mixture was gently swirled. The flask was immediately brought to a boil on a hot plate until white fumes were observed, then removed from the heat. Then, 10 mL of deionized water was added once the sample was cooled down to room temperature. Afterwards, the mixture was heated on the hot plate until the sample was dissolved, and then removed from the heat to cool. Afterwards, 100 mL of deionized water was added, and the mixture was shaken to homogenize. Finally, the mixture was filtered and analyzed using the Dual View Optical Emissions Spectrometer (Optima 8300, PerkinElmer, Inc., Waltham, MA, USA). Standard curves were implemented, and the conditions of the equipment were set as recommended by the manufacturer.

Carbohydrates were determined by difference once all other macronutrients were obtained [24] using the following Formula (4):

Carbohydrates (%) = 100 − [Fat (%) + Protein (%) + Moisture (%) + Ash (%)](4)

### 2.4. Preparation of Cookie Containing Cantaloupe Melon Seed Flour

Gluten-free cookies were prepared using a procedure similar to that described by Martinez et al. [25]. First, all the ingredients (Table 1) were weighed. Next, melon seed flour, rice flour (Great Value^TM^, Bentonville, AR, USA), butter (Great Value^TM^), stevia (Great Value^TM^), egg (Great Value^TM^), baking powder (Great Value^TM^), sugar (Great Value^TM^), dried cranberry (Ocean Spray, Middleborough, MA, USA), and vanilla (Molina, Guadalajara, Mexico) were placed and well mixed (90 s) in a stainless steel mixer (Globe, SP05-MIXER5QT, Dayton, OH, USA) attached with a spiral dough hook. Then, the cookie dough was manually shaped into a cylinder with a diameter of 3.80 ± 0.17 cm and cut into pieces with a thickness of 0.80 ± 0.05 cm. Subsequently, cookies were placed on a tray covered by parchment paper to avoid stickiness and baked in a preheated oven at 177 °C for 18 min (based on preliminary studies). Finally, the cookies were cooled and stored in resealable plastic bags until further use.

### 2.5. Physicochemical Characteristics of Cookies Containing Melon Seed Flour

#### 2.5.1. Color and Water Activity

Color (L*, a*, and b*) was determined at the center of the gluten-free cookie piece with the method already described above. Likewise, water activity (a_W_) was determined as already described above.

#### 2.5.2. Spread Factor

The spreading factor, defined as the ratio of diameter to thickness (D/T), was measured after baking, as reported by [26].

#### 2.5.3. Hardness

Texture analysis was carried out as described by Rivera et al. [27]. A texture analyzer (TA.XTplus^®^, Stable Micro Systems, Godalming, UK) with a TA-43 (knife blade of 9.6 cm height, 6.9 cm width, and 3 mm thick flat end bell lock) probe with a 50-kg load cell in a cut test setup. The conditions of the experiment were a test speed of 2.20 mm/s, a pre-test speed of 1.6 mm/s, a trigger force of 30 g, and a post-test speed of 12 mm/s. The distance of the experiment was set at 10 mm, and the results were expressed in newtons.

### 2.6. Consumer Acceptance of the Gluten-Free Cookies Containing Cantaloupe Melon Seed Flour

This research involving human subjects was approved by the Louisiana State University Agricultural Center Institutional Review Board (IRBAG-21-0063; Baton Rouge, LA, USA). The exclusion criteria were people under 18 years old, pregnant women, and those who were allergic to the food ingredients used in this study. A consent form was e-signed by all participants (N = 90) to acknowledge the potential minimal risk associated with food allergens presented in this study. Of these 90 participants, females accounted for 52% and males for 48%. In terms of age, 76% were in the range of 18–25 years, 19% in the 26–35 years, and 6% were 36 years or older. The racial composition was represented mainly by white/Caucasian, Latino, black or African American, and Asian, with 32, 24, 24, and 11%, respectively. The rest (8%) were identified with other races.

The participants were provided with five coded treatments of cookies (20, 40, 60, 80, and 100% of cantaloupe melon seed flour in substitution of rice flour) on a plastic tray along with napkins. Water and unsalted crackers were served to cleanse their palate between samples. They were asked to rate the acceptability of appearance, aroma, flavor, overall texture, grittiness, and overall liking using a 9-point hedonic scale (1 = extremely dislike, 5 = neither like nor dislike, 9 = extremely like). Additionally, their purchase intent was assessed before and after a sustainability claim about the use of cantaloupe melon seeds. It is important to highlight that before the consumer study, microbial tests for total coliforms, *E. coli*, total aerobic plate count, mold, and yeast were conducted to ensure the microbial safety of the final product for consumption.

### 2.7. Statistical Analysis

Data were analyzed using Statistical Analysis System (SAS^®^ version 9.4) with PROC GLM and PROC ANOVA. Means and their corresponding standard deviations were reported. All the experiments were conducted in triplicate. The physicochemical characteristics of the cookies (color, water activity, spread factor, and texture) were analyzed as a completely randomized design with PROC GML to determine differences (*p* < 0.05) with a post hoc Tukey test. Consumer acceptance data were analyzed using PROC ANOVA to find differences (*p* > 0.05). Moreover, the McNemar’s test was used to detect differences (*p* < 0.05) in purchase intent before and after a sustainability claim regarding the use of cantaloupe melon seed flour in cookies.

## 3. Results and Discussion

### 3.1. Physicochemical Characteristics of Cantaloupe Melon Seed Flour

#### 3.1.1. Water Activity (a_W_) and Color

Because the cantaloupe seeds were dehydrated for 12 h at 60 ± 1 °C before grinding, the flour’s a_W_ was expected to be low. Similar results were obtained by Araújo et al. [28] who reported a_W_ between 0.27 and 0.33 for germinated cantaloupe flour, while in this study, the a_W_ was 0.4 (Table 2).

The cantaloupe melon seed flour was light brown; the L*a*b* values are shown in Table 2. Our results are similar to those reported by Akbaş et al. [29] for melon seed powder (L* = 71.74, a* = 7.28, and b* = 29.13). The color attributes of melon seed flour are mainly due to its bioactive compounds, such as flavonoids and tannins [30], which may differ by variety [31].

#### 3.1.2. Proximate Composition and Mineral Profile of Cantaloupe Melon Seed Flour

The proximate composition of the cantaloupe melon seed flour (Table 3) was consistent with that reported for other melon seed flours. For example, for different types of melon in Bangladesh, Refat et al. [32] reported carbohydrate levels ranging from 46 to 66%, whereas in this study, it was 44.48% (Table 3). Moreover, the range of protein was between 16% and 29% whereas in this study it was 21.4%. Another study using the same type of melon [17] reported fiber and ash contents of 35% and 4%, respectively, which were practically similar to those found in this study (34.93% and 3%).

Magnesium, phosphorus, and potassium were the most prevalent minerals in this product. Similar results were observed by Ogunbusola et al. [33] in defatted and full-fat white melon seed flour, where magnesium and potassium, along with nickel, were the most abundant minerals. Based on Table 3, the fiber and protein content, along with its good mineral content, make cantaloupe melon seed flour a potential raw material for further processed foods.

### 3.2. Physicochemical Characteristics of Cookies Containing Cantaloupe Melon Seed Flour

#### 3.2.1. Color

Color lightness (L*) values decreased significantly (*p* < 0.05) with increasing cantaloupe melon seed flour, with T20 being the lightest with a value of 67.6 (Table 4). Meanwhile, T100 was more reddish (a* = 12.06) than T60, T40, and T20 samples (6.89–10.5). Similar results were reported by Al Masoud et al. [34], who found decreased L* values when muskmelon seed flour was incorporated into biscuits. According to Martins et al. [35], when reducing sugars interact with free amino acids, they can form brown, aromatic compounds called melanoidins. In this experiment, the addition of more melon seed flour increased protein content and, consequently, amino acid availability. This could explain the gradual decrease in L* values and the increase in a* values of cookies as the amount of melon seed flour increased. On the other hand, b* values (33.02–35.01) remained the same (Table 4).

#### 3.2.2. Spread Factor

The average spread factor of the unbaked cookie was 4.7 (1.5 cm/0.3 cm) ± 0.3. It can be observed that the spread factor significantly (*p* < 0.05) increased with increased cantaloupe melon seed flour, from 5.61 to 7.4 (Table 4). T100 samples had about 1.3–1.6 times the spread factor of T20 and unbaked cookies. One possible reason for the observed increase in the spread factor was the fat content in the cookies. Pareyt et al. [36] observed this trend, finding that spread factors were higher in sugar-snap cookies with higher fat content. In accordance with Panghal et al. [37], fat in the cookie structure prevents the gluten network from forming and melts during baking, thereby increasing the spread factor. They further reported that increasing fat content in cookie dough (up to 46.68%) resulted in cookies with a higher spread factor. In this study, increasing cantaloupe melon seed flour increased the fat content of the cookies, hence a greater spread factor (Table 4). This characteristic is important because the surface area increases with a higher spread factor, which also affects the color. Results from Table 4 indicate opposite trends for color lightness (L*) and spread factor values.

#### 3.2.3. Water Activity

As observed in Table 4, there was an inverse relationship between the spread factor and water activity; that is, the higher the spread factor, the lower the a_W_ values. This relationship may be attributed to the larger surface area of the cookie, which enhances heat exposure during baking, leading to increased moisture evaporation and a subsequent decrease in water activity. The results of this study align with those of Chikpah et al. [38], who discovered that cookies with a higher spread factor had the lowest moisture and water activity. Moreover, another factor that might decrease the a_W_ is its fiber content. In this study, the treatment with the highest fiber content was T100 due to the fiber content in melon seed flour. These results were corroborated by Grzelczyk et al. [39], who reported that the high-fiber cookies prepared from bamboo flour had reduced a_W_.

#### 3.2.4. Texture Hardness

The maximum force required to break cookies, expressed in newtons per treatment, is shown in Table 4. In this study, no statistical differences in hardness (N) were found among treatments (*p* > 0.05). In other studies, cookies with increased fat content showed lower hardness [36]. Moreover, gluten-free sugar-snap cookies with a lower spread factor showed higher hardness values [40].

### 3.3. Consumer Acceptance and Purchase Intent of the Gluten-Free Cookies Containing Cantaloupe Melon Seed Flour

According to the results in Table 4, the more melon seed flour was added, the darker (lower L* values) and the flatter the cookies were. These two main aspects of appearance (color and spread factor) may explain the lower acceptance scores for appearance observed at increasing melon seed flour levels (Table 5). However, even at 100% melon seed flour, the cookie’s appearance was still acceptable (score of 5.72). Similar results were reported by Pestorić et al. [41], who evaluated cookies with added medicinal herbs and found that people preferred the less brown cookies. This trend was also observed for aroma and flavor, with lower acceptance scores at higher flour concentrations. On the other hand, the opposite trend occurred for overall texture and grittiness, with treatments (T40–T100) containing more melon seed flour showing higher scores (*p* < 0.05) than T20. This suggests that consumers like crispier cookies. Finally, overall liking was directionally higher in the three middle concentrations, with T40 being the highest, with a score of 6.25. Statistical differences (*p* < 0.05) were found between the T40 and T100. Overall, the inclusion of melon seed flour, even at 100% (T100), did not cause the cookie products to be unacceptable (a score of 5.5).

Results for purchase intent before and after a sustainability claim are shown in Table 6. For cookies with 40% melon seed flour, the purchase intent before the claim was above 50%. After the claim, the change in purchase intent was statistically greater (*p* > 0.05) for T20, with an increase of 19%. As Cedillos et al. [42] found in their study on frozen yogurt with hesperidin added that consumers positively changed their opinion on purchase intent when additional information was provided.

## 4. Conclusions, Recommendations, and Future Studies

The cantaloupe melon seed flour produced in this study showed a light brownish color and served as a good source of protein (21.4%) and crude fiber (34.93%), which can be incorporated in various products. Baked cookies with more melon seed flour were flatter (increased spread factor) and browner in color, while water activity decreased. Consumers tentatively liked the cookie sample with 40% melon seed flour than the others. Finally, the sustainability claim (i.e., the use of byproducts) positively changed the purchase intent. Sustainability claims are an important tool when marketing value-added products prepared from food processing byproducts. This study demonstrated that cantaloupe melon seeds can be incorporated into the food system, potentially benefiting economic, environmental, and health outcomes.

Some limitations of this study were the small batch size in cookie production and the lack of equipment to simulate commercial-scale production. Standardizing the cookie production process using commercial equipment and increasing the batch size would help to better understand the dough behavior during large-scale production. It is recommended that future studies include a control treatment containing no melon seed flour (100% rice flour) to better understand the specific contribution of melon seed flour to the traditional formulation. Additionally, extending physicochemical analyses to include measurements of antioxidant capacity and activities, as well as bioactive compounds, in both melon seed flour and cookies is suggested. A comparative sensory evaluation of commercially available gluten-free cookies (prepared with gluten-free flours) could provide a broader perspective on the formulation developed in this study. Due to the high fat content in this cookie, an oxidative stability and shelf-life study is recommended.

## Figures and Tables

**Table 1:** Formulations of gluten-free cookies containing cantaloupe melon seed flour.

Ingredients (%)	Treatments *				
	T20	T40	T60	T80	T100
Cantaloupe Seed Flour	9.54	19.09	28.63	38.17	47.71
Rice Flour	38.17	28.63	19.09	9.54	0.00
Butter	19.61	19.61	19.61	19.61	19.61
Sugar	10.20	10.20	10.20	10.20	10.20
Stevia	0.97	0.97	0.97	0.97	0.97
Dried Cranberry	5.23	5.23	5.23	5.23	5.23
Vanilla	1.38	1.38	1.38	1.38	1.38
Baking Powder	0.52	0.52	0.52	0.52	0.52
Egg	14.38	14.38	14.38	14.38	14.38
Total	100.00	100.00	100.00	100.00	100.00

* T20, T40, T60, T80, and T100 = Cookies formulated, respectively, with 20, 40, 60, 80, and 100% by weight of the cantaloupe melon seed flour in substitution of rice flour.

**Table 2:** Color parameters and water activity of cantaloupe melon seed flour.

Parameter	Cantaloupe Melon Seed Flour
L* (lightness)	70.7 ± 0.5
a* (redness)	6.12 ± 0.13
b* (yellowness)	32.6 ± 1.6
Water activity	0.4 ± 0.02

**Table 3:** Macronutrients and mineral profile of cantaloupe melon seed flour.

Parameter	Cantaloupe Melon Seed Flour
Macronutrient (g/100 g)	
Protein	21.4 ± 0.8
Fat	26.9 ± 2
Moisture	4.0 ± 0.2
Total Ash	3 ± 0.0
Carbohydrates	44.48 ± 1.6
Crude Fiber	34.93 ± 1.7
Mineral Composition (mg/100 g)	
Boron	4.6 ± 1.2
Calcium	44.5 ± 6
Copper	2.3 ± 0.1
Iron	9.1 ± 0.2
Magnesium	387 ± 9
Manganese	2.9 ± 0.1
Phosphorus	770 ± 14
Potassium	755 ± 44
Sodium	48 ± 10
Sulphur	204 ± 12
Zinc	7.2 ± 0.1

**Table 4:** Selected physical characteristics of cookies containing different levels of cantaloupe melon seed flour.

Characteristics	Treatments ^a^				
	T20	T40	T60	T80	T100
L*	67.6 ± 4 ^A^	63.66 ± 1.3 ^AB^	61.0 ± 0.6 ^B^	59.65 ± 1.9 ^B^	58.5 ± 3 ^B^
a*	6.89 ± 1.1 ^D^	8.7 ± 0.2 ^C^	10.5 ± 0.6 ^B^	11.5 ± 0.7 ^AB^	12.06 ± 1.2 ^A^
b*	33.02 ± 1.2 ^A^	34.43 ± 1.2 ^A^	33.9 ± 1.0 ^A^	34.7 ± 0.8 ^A^	35.01 ± 1.5 ^A^
a_W_	0.5 ± 0.1 ^A^	0.4 ± 0.0 ^AB^	0.3 ± 0.0 ^B^	0.4 ± 0.1 ^B^	0.3 ± 0.0 ^B^
Spread Factor	5.6 ± 0.2 ^C^	5.8 ± 0.2 ^C^	6.2 ± 0.3 ^BC^	6.5 ± 0.3 ^B^	7.4 ± 0.2 ^A^
Hardness (N)	9 ± 3 ^A^	6.2 ± 1.4 ^A^	5.6 ± 1.3 ^A^	6.3 ± 2 ^A^	6.6 ± 1.0 ^A^

^a^ T20, T40, T60, T80, and T100 = Cookies formulated, respectively, with 20, 40, 60, 80, and 100% weight of the cantaloupe melon seed flour in substitution of rice flour. ^A,B,C,D^ means ± standard deviations in each row without a common letter are significantly (*p* < 0.05) different.

**Table 5:** Consumer acceptance of gluten-free cookies containing cantaloupe melon seed flour.

Attributes	Treatments *				
	T20	T40	T60	T80	T100
Appearance	6.94 ± 1.4 ^A^	6.94 ± 1.2 ^A^	6.28 ± 1.5 ^B^	6.25 ± 1.6 ^B^	5.72 ± 1.9 ^B^
Aroma	7.07 ± 1.4 ^A^	7.10 ± 1.2 ^A^	6.58 ± 1.4 ^AB^	6.21 ± 1.5 ^BC^	5.85 ± 1.8 ^C^
Flavor	6.08 ± 1.8 ^AB^	6.54 ± 1.7 ^A^	6.08 ± 1.7 ^AB^	6.26 ± 1.6 ^AB^	5.65 ± 1.9 ^B^
Overall Texture	5.01 ± 1.9 ^B^	6.11 ± 1.7 ^A^	6.18 ± 1.6 ^A^	6.18 ± 1.6 ^A^	5.83 ± 1.8 ^A^
Grittiness	4.45 ± 1.9 ^B^	5.76 ± 1.7 ^A^	5.90 ± 1.7 ^A^	5.86 ± 1.7 ^A^	5.51 ± 1.8 ^A^
Overall Liking	5.63 ± 1.7 ^AB^	6.25 ± 1.7 ^A^	6.08 ± 1.5 ^AB^	5.98 ± 1.6 ^AB^	5.50 ± 1.9 ^B^

* T20, T40, T60, T80, and T100 = Cookies formulated, respectively, with 20, 40, 60, 80, and 100% by weight of the cantaloupe melon seed flour in substitution of rice flour. ^A,B,C^ means ± standard deviations in each row not containing a common letter are significantly (*p* < 0.05) different.

**Table 6:** Purchase intent (frequency) of the gluten-free cookies containing cantaloupe melon seed flour before and after a sustainability claim.

Treatment *	Purchase Intent Before a Sustainability Claim		Purchase Intent After a Sustainability Claim		Pr > F **
	No	Yes	No	Yes	
T20	61	29	42	48	<0.0001
T40	32	58	33	57	0.7389
T60	47	43	41	49	0.0833
T80	43	47	41	49	0.3173
T100	52	38	48	42	0.1573

* T20, T40, T60, T80, and T100 = Cookies formulated, respectively, with 20, 40, 60, 80, and 100% by weight of the cantaloupe melon seed flour in substitution of rice flour. ** For each treatment, the increase in the “yes” response after vs. before a sustainability claim was significant at *p* < 0.05.

## Data Availability

The original contributions presented in this study are included in this article. Further inquiries can be directed to the corresponding author.
